# Polynucleotide Mixture Attenuates Ultraviolet B-Induced Skin Pigmentation

**DOI:** 10.3390/ijms26136399

**Published:** 2025-07-03

**Authors:** Seyeon Oh, Hee-Dae Jeon, Nark-Kyoung Rho, Kuk Hui Son, Kyunghee Byun

**Affiliations:** 1 Functional Cellular Networks Laboratory, Lee Gil Ya Cancer and Diabetes Institute, Gachon University, Incheon 21999, Republic of Korea; 2 Banobagi Dermatology Clinic, Seoul 06109, Republic of Korea; 3 Leaders Aesthetic Laser & Cosmetic Surgery Center, Seoul 06014, Republic of Korea; 4 Department of Thoracic and Cardiovascular Surgery, Gachon University Gil Medical Center, Gachon University, Incheon 21565, Republic of Korea; 5 Department of Anatomy & Cell Biology, College of Medicine, Gachon University, Incheon 21936, Republic of Korea; 6 Department of Health Sciences and Technology, Gachon Advanced Institute for Health & Sciences and Technology (GAIHST), Gachon University, Incheon 21999, Republic of Korea

**Keywords:** polynucleotide, NLRP3 inflammasome, melanogenesis, skin pigmentation

## Abstract

Ultraviolet (UV) radiation stimulates melanogenesis, leading to various esthetic problems. UV increases oxidative stress and nuclear factor-kappa B (NF-κB), which increase the nucleotide-binding oligomerization domain (NOD) or leucine-rich repeat and pyrin do-main containing 3 (NLRP3) inflammasome. Given that polydeoxyribonucleotides reduce melanogenesis and polynucleotide (PN) has molecular similarity to polydeoxyribonucleotides, we hypothesized that PN can decrease melanogenesis. We compared the anti-melanogenic effect of PN with that of a PN mixture (PNM) that contained other antioxidants, such as glutathione and hyaluronic acid, in UVB-irradiated keratinocytes and animal skin. PN and PNM both decreased oxidative stress, which was evaluated according to the expression of NADPH oxidase (NOX) 1/2/4, the glutathione (GSH):oxidized glutathione (GSSG) ratio, and 8-hydroxy-2′-deoxyguanosine (8-OHdG) in UVB-irradiated keratinocytes. The expression of NLRP3 inflammasome components (NLRP3, ASC, and pro-caspase-1) and IL-18 was increased by UVB radiation and reduced by PN and PNM. When conditioned media from PN or PNM were administered to UVB-radiated keratinocytes, melanogenesis-related signals (MITF, tyrosinase, and tyrosinase-related protein1/2) were decreased. These effects were similar in the UVB-irradiated animal skin. Both PN and PNM decreased melanin accumulation and increased skin lightness in UVB-irradiated skin. The anti-melanogenic effect of PNM was greater than that of PN. In conclusion, PN and PNM decreased melanogenesis by decreasing oxidative stress, NF-κB, and NLRP3 inflammasome activation.

## 1. Introduction

Reactive oxygen species (ROS) are the primary cause of ultraviolet (UV)-induced photodamage in the skin [1]. NADPH oxidase (NOX) enzymes generate intracellular ROS, which are activated by various cellular stresses, including UV exposure [2]. Cells have ROS removal systems, such as glutathione peroxidase (GPX), superoxide dismutase (SOD), and glutathione (GSH) [3]. Disruption of the balance between ROS generation and removal exposes cells to various oxidative stress-related injuries. Long-term exposure of the skin to UV radiation increases ROS, which can act as second messengers and upregulate various signaling pathways, such as nuclear factor-kappa B (NF-κB) [4].

UV radiation also triggers DNA damage by increasing nucleotide-binding oligomerization domain (NOD) or leucine-rich repeat and pyrin domain containing 3 (NLRP3) inflammasome formation in keratinocytes [5].

During NLRP3 inflammasome activation, NF-κB initiates a priming step by increasing NLRP3 expression [6,7]. The second step is activation, which increases the assembly of inflammasomes and the maturation of interleukin (IL)-1β and IL-18 [7]. NLRP3 inflammasomes are composed of NLRP3, an apoptosis-associated, speck-like protein containing a caspase-recruitment domain (ASC) and pro-caspase-1 [8]. After assembly, pro-caspase-1 is cleaved into the active form, caspase-1, which cleaves pro-IL-1β and pro-IL-18 into their active forms, IL-1β and IL-18, respectively [7]. Caspase-1 also cleaves gasdermin D (GSDMD), which generates an N-terminal domain (NT-GSDMD) [9]. NT-GSDMD forms pores on the cell membrane, which induces pyroptosis and the release of mature IL-1β and IL-18 into the extracellular space [9,10]. These cytokines increase tissue inflammation. IL-18 increases melanogenesis by upregulating various signaling pathways, such as mitogen-activated protein kinase (MAPK) and protein kinase C (PKC), which activate transcription factor microphthalmia-associated transcription factor (MITF) [11,12].

Melanin is generated in the skin via the stimulation of MITF, which activates downstream pathways, such as tyrosinase, tyrosinase-related protein 1 (TRP1), and tyrosinase-related protein 2 (TRP2) [13]. Melanin acts as the main protector of the skin against UV radiation [14]; however, excessive production of melanin leads to various esthetic problems, such as melasma, freckles, post-inflammatory hyperpigmentation, and senile lentigines [15].

Polydeoxyribonucleotide (PDRN) is a DNA fragment that is isolated from the sperm cells of salmon trout and chum salmon; its molecular weight varies between 50 and 1500 kDa [16]. PDRN has been used for skin rejuvenation owing to its anti-inflammatory effects and ability to promote collagen synthesis [17]. PDRN inhibits NF-κB and inflammatory cytokines, such as IL-6 [18]. It also decreases melanogenesis by decreasing MITF and tyrosinase activity [19]. However, the exact mechanism by which PDRN decreases tyrosinase activity has not been fully elucidated [17]. Polynucleotide (PN) has been isolated from the testes of Pacific salmon, rainbow trout, and sturgeons [20]. The molecular weight of PN is higher than that of PDRN (8000 kDa) [21]. Because PN is viscoelastic, it can provide a scaffold structure [22]. PN also has anti-inflammatory effects and increases collagen synthesis [23,24]. The exact mechanism by which PN exerts these effects has not yet been fully elucidated. Because PN is molecularly similar to PDRN, it is postulated that their mechanisms of action are similar [25].

Although PDRN exhibits an inhibitory effect on melanogenesis, its anti-melanogenic effect has not been fully evaluated. Thus, we evaluated whether PN could decrease melanogenesis using in vitro and UVB-irradiated animal models. We also compared the effect of PN on melanogenesis with that of a PN mixture (PNM) containing hyaluronic acid, glycerin, GSH, and PN. Moreover, we compared the anti-melanogenic effect of PN with that of a PNM containing an antioxidant mixture (AM). We hypothesized that PN could reduce ROS levels, in turn decreasing NF-κB activity and NLRP3 inflammasome activation. By reducing the NLRP3 inflammasome, IL-18 secretion was decreased, which eventually inhibited melanogenesis-related signaling pathways, such as MITF/Tyrosinase/TRP1/TRP2, and decreased skin melanin.

## 2. Results

### 2.1. PNM, PN, and AM Decreased Oxidative Stress and NF-κB in UVB-Irradiated Keratinocytes

The cytotoxic effects of varying concentrations of PNM (0.044–22 mg/mL), which contained hyaluronic acid, glycerin, GSH, and PN (Table S1), on human keratinocytes (HaCaT) were assessed after 24 h of incubation. No cytotoxicity was observed at concentrations of up to 13.2 mg/mL of PNM (Figure S1).

The ratio of reduced GSH to oxidized glutathione (GSSG) is frequently used as a marker of oxidative stress [26]. UVB-irradiated keratinocytes were used to identify the effective concentration of PNM that reduced oxidative stress. UVB radiation decreased the GSH:GSSG ratio, whereas PNM increased the GSH:GSSG ratio. The increase in the GSH:GSSG ratio was not substantially different at 2.2, 4.4, and 6.6 mg/mL of PNM. Thus, 2.2 mg/mL of PMN was identified as a suitable concentration for subsequent experiments (Figure S2).

**Figure 1:**
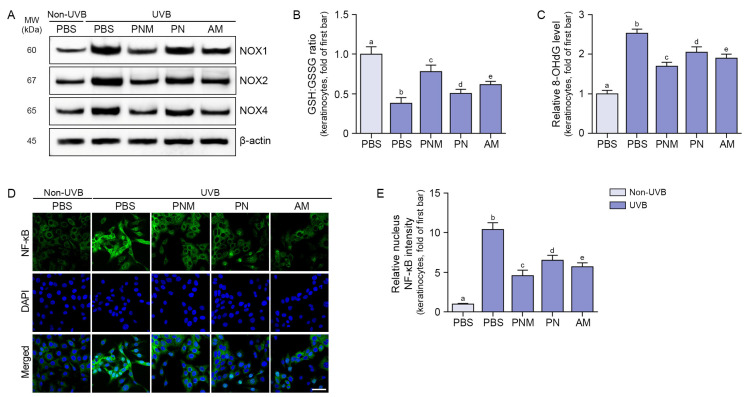
Reduction in oxidative stress and NF-κB translocation by PNM, PN, or AM in UVB-irradiated keratinocytes. (**A**) Western blot analysis showing the protein expression levels of NOX1/2/4 in keratinocytes exposed to UVB and treated with PNM, PN, or AM. UVB irradiation increased NOX1/2/4 expression, while PNM, PN, or AM treatment reduced their levels. β-actin was used as a loading control. The quantification of Western blot bands is provided in Figure S3. (**B**) The GSH:GSSG ratio was measured to evaluate the antioxidative effects of PNM, PN, and AM in UVB-exposed keratinocytes using a GSH/GSSG-Glo assay kit. UVB irradiation significantly decreased the GSH:GSSG ratio, while PNM, PN, or AM treatment restored it. (**C**) Relative levels of 8-OHdG were analyzed to assess the regulation of oxidative stress by PNM, PN, and AM in UVB-exposed keratinocytes using ELISA. UVB irradiation significantly increased 8-OHdG levels, whereas PNM, PN, and AM treatments reduced oxidative DNA damage. (**D**) ICC staining showing the effect of PNM, PN, and AM on NF-κB translocation in UVB-exposed keratinocytes. NF-κB is shown in green and nuclei are counterstained with DAPI (blue). Scale bar = 50 μm. (**E**) Quantification of NF-κB localization in the nucleus from ICC images, showing that PNM, PN, and AM treatments each reduce UVB-induced NF-κB nuclear translocation. Data are expressed as the mean ± SD from three independent experiments. Statistical significance was set at *p* < 0.05. Groups sharing the same letter (a–e) are not significantly different based on multiple comparisons using the Mann–Whitney U test. AM, antioxidant mixture; DAPI; 4′,6-diamidino-2-phenylindole; ELISA, enzyme-linked immunosorbent assay; GSH, glutathione; GSSG, glutathione disulfide; ICC, immunocytochemistry; MW, molecular weight; NF-κB, nuclear factor-kappa B; NOX, NADPH oxidase; PBS, phosphate-buffered saline; PN, polynucleotides; PNM, PN mixture; SD, standard deviation; UVB, ultraviolet B; 8-OHdG, 8-hydroxy-2′-deoxyguanosine.

The expression of NOX1/2/4 was increased by UVB irradiation and decreased by PNM, PN, and AM treatments. This decrease was more pronounced in the PNM group than in the PN or AM group (Figure 1A and Figure S3).

The GSH:GSSG ratio was decreased by UVB irradiation and increased by PNM, PN, and AM treatments. This effect was more prominent in the PNM group than in the PN or AM group (Figure 1B). The widely used oxidative stress biomarker 8-hydroxy-2′-deoxyguanosine (8-OHdG) [27] was increased by UVB irradiation and decreased by PNM, PN, and AM treatments. This effect was more prominent in the PNM group than in the PN or AM group (Figure 1C).

The activity of NF-κB was evaluated by examining its translocation into the nucleus. The translocation of NF-κB was increased by UVB radiation and decreased by PNM, PN, and AM treatments. This decrease was more prominent in the PNM group than in the PN or AM group (Figure 1D,E).

### 2.2. PNM, PN, and AM Decreased NLRP3 Inflammasomes and IL-18 in UVB-Irradiated Keratinocytes

**Figure 2:**
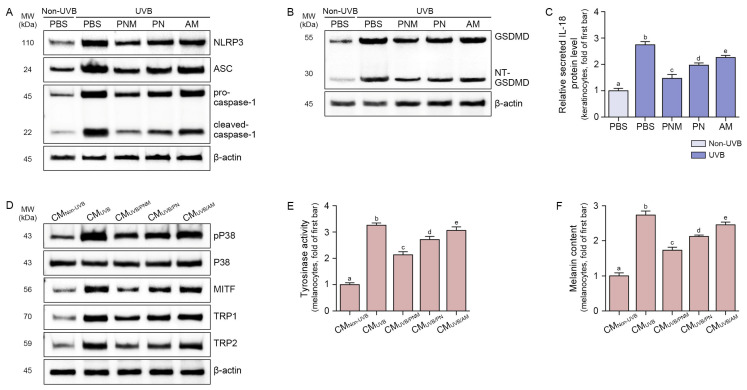
Suppression of NLRP3 inflammasome activation, pyroptosis, and melanogenesis by PNM, PN, or AM in UVB-irradiated keratinocytes. (**A**) Representative Western blot images showing the protein expression levels of NLRP3, ASC, pro-caspase-1, and cleaved-caspase-1 in keratinocytes exposed to UVB and treated with PNM, PN, or AM. Treatment with PNM, PN, or AM reduced UVB-induced NLRP3 inflammasome activation. The quantification of Western blot bands is provided in Figure S4A–D. (**B**) Western blot analysis of GSDMD and NT-GSDMD protein expression in UVB-irradiated keratinocytes treated with PNM, PN, or AM, indicating suppression of pyroptosis. The quantification of Western blot bands is provided in Figure S4E. β-actin was used as a loading control. (**C**) ELISA quantification of IL-18 secretion in the culture supernatant of UVB-irradiated keratinocytes, showing that treatment with PNM, PN, or AM reduced UVB-induced IL-18 levels. (**D**) Western blot analysis of pP38, P38, MITF, TRP1, and TRP2 in B16F10 melanoma treated with CM collected from non-UVB- or UVB-irradiated HaCaT keratinocytes pretreated with PNM, PN, or AM. CM from UVB-exposed keratinocytes increased melanogenesis-related protein expression, which was attenuated by PNM, PN, or AM treatment. The quantification of Western blot bands is provided in Figure S5. β-actin was used as a loading control. (**E**,**F**) Quantification of tyrosinase activity (**E**) and melanin content (**F**) in B16F10 cells treated with CM from non-UVB- or UVB-irradiated HaCaT keratinocytes, showing that PNM, PN, and AM all reduced UVB-induced melanogenesis. Values represent the mean ± SD of three independent experiments. Statistical comparisons were performed using the Mann–Whitney U test, with *p* < 0.05 considered significant. Identical letters (a–e) denote no statistically significant difference between the respective groups. AM, antioxidant mixture; ASC, apoptosis-associated speck-like protein containing a CARD; CM, conditioned media from keratinocyte; CM_Non-UVB_, conditioned media from the non-UVB/PBS group of keratinocytes; CM_UVB_, conditioned media from the UVB/PBS group of keratinocytes; CM_UVB/PN_, conditioned media from the UVB/PN group of keratinocytes; CM_UVB/PNM_, conditioned media from the UVB/PNM group of keratinocytes; ELISA, enzyme-linked immunosorbent assay; GSDMD, gasdermin D; IL-18, interleukin-18; MITF, microphthalmia-associated transcription factor; MW, molecular weight; NLRP3, NOD-, LRR-, and pyrin domain-containing protein 3; NT-GSDMD, N-terminal GSDMD; PBS, phosphate-buffered saline; pP38, phosphorylated P38; PN, polynucleotide; PNM, PN mixture; SD, standard deviation; TRP, tyrosinase-related protein; UVB, ultraviolet B.

The expression of NLRP3 inflammasome components (NLRP3, ASC, and pro-caspase-1) and cleaved-caspase-1 was increased by UVB irradiation and decreased by PNM, PN, and AM. This decrease was more prominent in the PNM group than in the PN or AM group (Figure 2A and Figure S4A–D).

The NT-GSDMD:GSDMD ratio increased with UVB irradiation and decreased with PNM, PN, and AM treatment. This decrease was more prominent in the PNM group than in the PN or AM group (Figure 2B and Figure S4E).

The secretion of IL-18 from keratinocytes was increased by UVB radiation and decreased using PNM, PN, and AM. This decrease was more prominent in the PNM group than in the PN or AM group (Figure 2C).

### 2.3. PNM, PN, and AM Decreased Melanogenesis-Related Signals in the Melanocyte

Since UV light affects keratinocytes first, which then promote melanocytes to increase melanogenesis, melanocytes were treated with conditioned media (CM) from UVB-irradiated keratinocytes.

The phosphorylated p38 (pP38):total P38 ratio was increased by CM from UVB-irradiated keratinocytes (CM_UVB_) and decreased by CM from UVB-irradiated keratinocytes treated with PNM (CM_UVB/PMN_), PN (CM_UVB/PN_), or AM (CM_UVB/AM_). This decrease was more prominent in the CM_UVB/PMN_ group than in the CM_UVB/PN_ or CM_UVB/AM_ group (Figure 2D and Figure S5A).

The expression of MITF/TRP1/TRP2 was increased by CM_UVB_ and decreased by CM_UVB/PMN_, CM_UVB/PN_, or CM_UVB/AM_. The decrease was more prominent in the CM_UVB/PMN_ group than in the CM_UVB/PN_ or CM_UVB/AM_ group (Figure 2D and Figure S5B–D).

Tyrosinase activity was increased by CM_UVB_ and decreased by CM_UVB/PMN_, CM_UVB/PN_, or CM_UVB/AM_. This decrease was more prominent in the CM_UVB/PMN_ group than in the CM_UVB/PN_ or CM_UVB/AM_ group (Figure 2E).

The melanin content was increased by CM_UVB_ and decreased by CM_UVB/PMN_, CM_UVB/PN_, or CM_UVB/AM_. This decrease was more prominent in the CM_UVB/PMN_ group than in the CM_UVB/PN_ or CM_UVB/AM_ group (Figure 2F).

### 2.4. PN and PNM Decreased Oxidative Stress and NLRP3 Inflammasome Activation in UVB-Irradiated Skin

**Figure 3:**
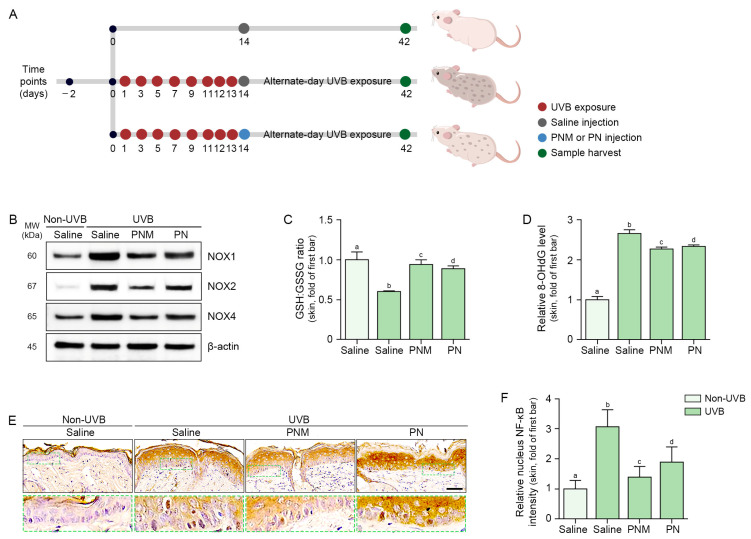
PNM- or PN-mediated reduction in oxidative stress and NF-κB translocation in UVB-irradiated mouse skin. (**A**) HRM-2 hairless mice (8-week-old, male) were exposed to UVB radiation on days 1, 3, 5, 7, 9, 11, 12, and 13 to induce skin pigmentation. On day 14, saline, PNM, or PN was intradermally injected. After injection, UVB exposure was continued every other day (alternate-day UVB exposure) until day 42 to maintain skin pigmentation. Skin samples were collected on day 42 for analysis. Red circles indicate UVB exposure, gray circles indicate saline injection, blue circles indicate PN or PNM injection, and green circles indicate sample harvesting. (**B**) Western blot analysis of NOX1/2/4 protein expression in UVB-irradiated mouse skin treated with PNM or PN. UVB irradiation increased NOX protein expression, while PNM or PN treatment attenuated these levels. β-actin was used as a loading control and the quantification of Western blot bands is provided in Figure S6. (**C**) Measurement of the GSH:GSSG ratio in UVB-irradiated mouse skin using the GSH/GSSG-Glo assay kit (GSH:GSSG ratio detection kit). The GSH:GSSG ratio in UVB-irradiated skin tissue decreased following UVB irradiation, but is restored by PNM or PN treatment. (**D**) ELISA quantification of 8-OHdG levels in UVB-exposed mouse skin treated with PNM or PN. Relative 8-OHdG levels in skin tissue, indicating increased oxidative DNA damage after UVB exposure. PNM or PN treatment significantly reduced 8-OHdG accumulation. (**E**) IHC staining of NF-κB translocation in UVB-irradiated mouse skin treated with PNM or PN. NF-κB is shown in brown and nuclei are counterstained with hematoxylin stain (blue). Scale bar = 50 μm. (**F**) Quantification of nuclear NF-κB intensity from IHC images, demonstrating that PNM and PN reduced UVB-induced NF-κB activation. Three independent experiments were conducted; data are shown as mean ± SD. Differences between groups were analyzed using the Mann–Whitney U test, with a threshold for significance set at *p* < 0.05. Groups marked with the same letters (a–d) are statistically indistinguishable. ELISA, enzyme-linked immunosorbent assay; GSH, glutathione; GSSG, glutathione disulfide; IHC, immunohistochemistry; MW, molecular weight; NF-κB, nuclear factor-kappa B; NOX, NADPH oxidase; PN, polynucleotide; PNM, PN mixture; SD, standard deviation; UVB, ultraviolet B; 8-OHdG, 8-hydroxy-2′-deoxyguanosine.

Since the effects of AM on decreased NLRP3 inflammasome activation and decreased melanogenesis-related protein were lower than those of PN or PNM, it was not used in the animal studies. Only PN and PNM were intradermally injected in the UVB-induced skin pigmentation model, and skin samples were collected 28 days later (Figure 3A).

The expression of NOX1/2/4 was increased by UVB radiation and decreased by PN and PNM in the UVB-irradiated skin. This decrease was more prominent in the PNM group than in the PN group (Figure 3B and Figure S6). The GSH:GSSG ratio was decreased by UVB irradiation and increased by the PN and PNM treatments. This increase was more prominent in the PNM group than in the PN group (Figure 3C). The expression of 8-OHdG was increased by UVB radiation and decreased by PN and PNM in UVB-irradiated skin. This decrease was more prominent in the PNM group than in the PN group (Figure 3D). Translocated NF-κB in the nucleus was increased by UVB radiation and decreased by PN and PNM. This decrease was more prominent in the PNM group than in the PN group (Figure 3E,F).

**Figure 4:**
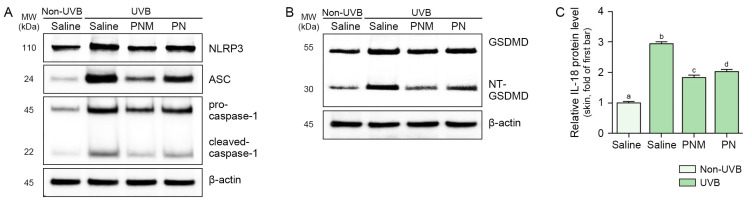
Suppression of NLRP3 inflammasome activation and pyroptosis by PNM or PN in UVB-irradiated mouse skin. (**A**) Representative Western blot images showing the protein expression levels of NLRP3, ASC, pro-caspase-1, and cleaved-caspase-1 in UVB-irradiated mouse skin and treated with PNM or PN. Treatment with PNM or PN reduced UVB-induced NLRP3 inflammasome activation. The quantification of Western blot bands is provided in Figure S7A–D. (**B**) Western blot analysis of GSDMD and NT-GSDMD protein expression in UVB-irradiated mouse skin and treated with PNM or PN, indicating suppression of pyroptosis. The quantification of Western blot bands is provided in Figure S7E. β-actin was used as a loading control. (**C**) ELISA quantification of IL-18 in UVB-irradiated skin, showing that PNM and PN both reduced UVB-induced IL-18 levels. All data are presented as mean ± SD from three biologically independent replicates. Statistical differences between groups were evaluated by the Mann–Whitney U test (*p* < 0.05). Letters (a–d) indicate statistical grouping; groups with the same letter are not significantly different. ASC, apoptosis-associated speck-like protein containing a CARD; ELISA, enzyme-linked immunosorbent assay; GSDMD, gasdermin D; IL-18, interleukin-18; MW, molecular weight; NLRP3, NOD-, LRR-, and pyrin domain-containing protein 3; NT-GSDMD, N-terminal GSDMD; PN, polynucleotide; PNM, PN mixture; SD, standard deviation; UVB, ultraviolet B.

The expression levels of NLRP3, ASC, pro-caspase-1, and cleaved-caspase-1 were increased by UVB irradiation and decreased by PN and PNM. This decrease was more prominent in the PNM group than in the PN group (Figure 4A and Figure S7A–D). NT-GSDMD:GSDMD was increased by UVB irradiation and decreased by PN and PNM treatments. This decrease was more prominent in the PNM group than in the PN group (Figure 4B and Figure S7E). The relative IL-18 protein level was increased by UVB radiation and decreased by PN and PNM treatments. This decrease was more prominent in the PNM group than in the PN group (Figure 4C).

### 2.5. PN and PNM Decreased Melanogenesis Signals and Melanin Accumulation in UVB-Irradiated Skin

**Figure 5:**
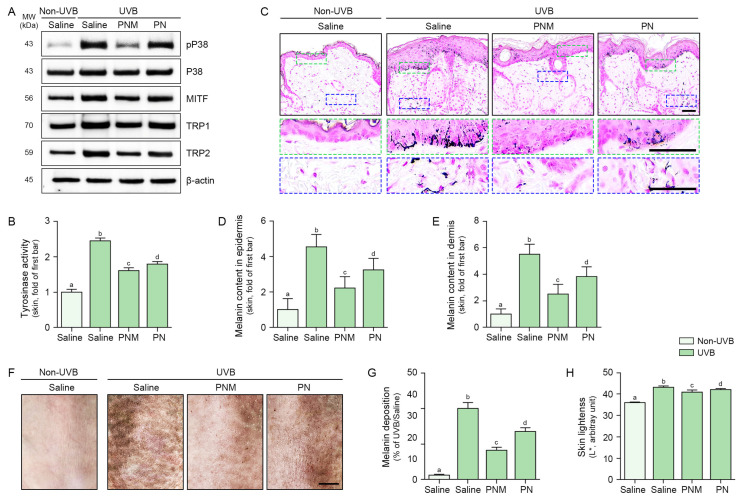
Reduction in melanogenesis by PNM or PN in UVB-irradiated mouse skin. (**A**) Western blot analysis showing the protein expression levels of pP38, P38, MITF, TRP1, and TRP2 in UVB-irradiated mouse skin treated with PNM or PN. UVB irradiation increased the expression of these melanogenesis-related proteins, whereas PNM or PN treatment reduced their levels. β-actin was used as a loading control. Quantification of Western blot bands is provided in Figure S8. (**B**) Tyrosinase activity was measured using the Tyrosinase Activity Kit. UVB exposure increased tyrosinase activity, which was suppressed by PNM or PN treatment. (**C**) Fontana–Masson staining of skin sections showing melanin content. UVB irradiation induced melanin accumulation in both the epidermis (green dotted box) and dermis (blue dotted box), which was attenuated by PNM or PN treatment. Scale bar = 50 µm. (**D**,**E**) Quantification of melanin content in the epidermis (**D**) and dermis (**E**) from Fontana–Masson staining images, showing that PNM or PN treatment significantly reduced UVB-induced melanin content. (**F**) Representative macroscopic images of skin pigmentation, showing increased UVB-induced hyperpigmentation, which was alleviated by PNM or PN treatment. Scale bar = 500 mm. (**G**) Quantification of melanin deposition on the skin surface from skin microscopic images, showing that PNM or PN treatment significantly reduced UVB-induced melanin deposition. (**H**) Skin lightness (L* value) quantification, demonstrating that UVB irradiation reduced skin brightness, while PNM or PN treatment restored it. All data are presented as mean ± SD from three biologically independent replicates. Statistical differences between groups were evaluated by the Mann–Whitney U test (*p* < 0.05). Letters (a–d) indicate statistical grouping; groups with the same letter are not significantly different. MITF, microphthalmia-associated transcription factor; MW, molecular weight; pP38, phosphorylated P38; PN, polynucleotide; PNM, PN mixture; SD, standard deviation; TRP, tyrosinase-related protein; UVB, ultraviolet B.

The pP38:total P38 ratio was increased by UVB radiation and decreased by PN and PNM in UVB-irradiated skin. This decrease was more prominent in the PNM group than in the PN group (Figure 5A and Figure S8A).

The expression of MITF/TRP1/TRP2 was increased by UVB irradiation and decreased by PN and PNM treatments. This decrease was more prominent in the PNM group than in the PN group (Figure 5A and Figure S8B–D).

Tyrosinase activity was increased by UVB radiation and decreased by PN and PNM treatments. This decrease was more prominent in the PNM group than in the PN group (Figure 5B).

The amount of melanin in the epidermis and dermis was increased by UVB radiation and decreased by the PN and PNM treatments. This decrease was more prominent in the PNM group than in the PN group (Figure 5C–E).

Melanin deposition on the skin surface was increased following UVB radiation and decreased by PN and PNM treatment, with a more pronounced reduction in the PNM group than in the PN group (Figure 5F,G).

Skin lightness, as evaluated using a colorimeter, was increased by UVB radiation and decreased by PN and PNM. This decrease was more prominent in the PNM group than in the PN group (Figure 5H).

## 3. Discussion

Both intrinsic and extrinsic factors, such as hormones and UV radiation, respectively, are involved in melanogenesis [28]. More than 150 genes are involved in controlling melanocyte differentiation, survival, and melanin generation during melanogenesis [21]. UV radiation results in alpha-melanocyte-stimulating hormone (α-MSH) secretion from keratinocytes, which binds to melanocortin 1 receptor (MC1R) on the melanocyte [29]. MC1R upregulates the cyclic adenosine monophosphate (cAMP)/cAMP response element-binding protein (CREB) signaling pathway, which increases the transcription of MITF [29].

Tyrosinase, the main enzyme involved in melanin synthesis, converts tyrosine into dopa, which in turn converts dopa into dopaquinone. Dopaquinone can be converted into glutathione (dopa) via conjugation with GSH. Dopaquinone is converted into pheomelanin or eumelanin. When cysteine is involved, dopaquinone is converted into pheomelanin, which is yellow to red in color [30]. In the absence of cysteine, TRP1, and TRP2 convert dopaquinone into eumelanin, which is brown to black [30].

Skin inflammation, which is caused by various factors including oxidative stress, also increases melanogenesis. IL-18 upregulates tyrosinase activity, MITF, TRP1, and TRP2 via activation of NF-κB in normal human foreskin-derived epidermal melanocytes (NHEMs) [11]. Advanced glycation end-products (AGEs) also increase melanogenesis by upregulating the NLRP3 inflammasome and IL-18 in fibroblasts [31].

PDRN inhibited NF-κB and inflammatory cytokines, which are upregulated by NF-κB [18]. PDRN also decreased testicular injury via decreasing NLRP3 inflammasome and IL-β [32].

Since melanogenesis is affected by NF-κB and NLRP3 inflammasomes, which could be decreased by PDRN, we hypothesized that PN could decrease melanogenesis by decreasing NF-κB and NLRP3 inflammasomes.

PNM contains hyaluronic acid, glycerin, GSH, and PN. Hyaluronic acid is frequently used as a biostimulating filler to increase collagen synthesis [33], but it also decreases UV-induced skin pigmentation [34]. Hyaluronic acid also shows antioxidant effects by increasing the removal of ROS via the upregulation of nuclear factor-erythroid-2-related factor 2 (Nrf2), which is the main transcription factor involved in cellular redox reactions [35].

GSH causes skin whitening by directly decreasing tyrosinase activity [36]. Moreover, GSH is indirectly involved in decreasing melanogenesis by removing ROS [37].

Since hyaluronic acid and GSH decrease melanogenesis, a more dramatic decrease in melanogenesis would be evident when hyaluronic acid and GSH are added to PN.

The results of this study showed that PN decreased oxidative stress, evidenced by decreased NOX1/2/4 and 8-OHdG and increased GSH:GSSG ratio in UVB-irradiated keratinocytes. Oxidative stress decreased with PN and PNM treatments. UVB irradiation also increased the translocation of NF-κB and NRLP3 inflammasome activation in keratinocytes. Furthermore, IL-18 levels were increased upon UVB irradiation. These levels were decreased by the PN and PNM treatments. When melanocytes were treated with the CM from UVB-irradiated keratinocytes, the expression of pP38/P38, MITF, TRP1, TRP2, and tyrosinase increased. These levels were decreased by the PN and PNM treatments. These changes were also shown in the AM-treated group; however, the effect of AM was lower than that of PN or PNM.

Similarly to the in vitro model, UVB-irradiated animal skin exhibited increased oxidative stress, NF-κB activity, NLRP3 inflammasome activation, and IL-18 expression. These levels were decreased by the PN and PNM treatments.

Because tyrosinase is a key enzyme in melanogenesis, many studies have been performed to identify tyrosinase inhibitors that decrease skin hyperpigmentation [38]. In fact, many tyrosinase inhibitors such as hydroquinone, arbutin, kojic acid, azelaic acid, ascorbic acid, and tranexamic acid have been used for skin whitening [39]. However, the use of these agents is associated with several complications. Hydroquinone can induce irritation, erythema, contact dermatitis, and ochronosis [40]. Arbutin is chemically unstable and has the potential to release benzene metabolites that suppress bone marrow [41]. Kojic acid has potential carcinogenicity and instability during storage; thus, it is no longer used in cosmetics [42]. Additionally, ascorbic acid is easily degraded by heat [43].

PDRN exhibited low toxicity even after systemic administration. The repeated systemic administration of 8 mg/kg was not toxic [44]. PDRN showed no toxicity when administered for 8 weeks via the intramuscular and perilesional routes in patients with diabetic ulcers [45]. A post-marketing surveillance study that evaluated 300,000 PDRN cases over five years showed excellent safety [16].

The safety of PN has not been as thoroughly evaluated as that of PDRN. However, injectable PN-based materials are safe, and the main reported complications are injection-related side effects, such as erythema or hematoma [46]. PN with hyaluronic acid also showed no toxicity in human or mouse fibroblast cells [47].

Although this study did not evaluate the long-term complications associated with PN or PNM, it is expected that PN or PNM would result in fewer complications than the topical use of previous tyrosinase inhibitors.

Photoaging also leads to increased expression of matrix metalloproteinases (MMPs), which destroys collagen or elastin fibers [48]. PDRN has also been reported to decrease MMP expression in fibroblasts [19]. Thus, it is also expected that PN could be beneficial in decreasing photoaging, including hyperpigmentation.

Our study showed that PNM had a more prominent effect on decreasing melanogenesis than PN or AM. To determine whether the effect of PNM exceeds the combined effects of PN and AM, that is, to assess the presence of a synergistic effect when PN and AM are combined, we employed the Bliss independence model, which is widely used for evaluating drug synergy [49]. In most cellular signaling pathways examined in this study, PNM exhibited a synergistic effect according to the Bliss independence model (Table S2).

It seems that the synergistic effect of decreasing the oxidative stress of hyaluronic acid and GSH with PN resulted in a greater decrease in the anti-melanogenic effect.

## 4. Materials and Methods

### 4.1. AM, PN, and PNM Preparation

AM, PN, and PNM were provided by RF Bio Co., Ltd. (Gangwon, Republic of Korea). The AM contained hyaluronic acid (10 mg/mL), glycerin (5 mg/mL), and GSH (1 mg/mL). The PN contained sodium polynucleotide (25 mg/mL) and lidocaine hydrochloride (3 mg/mL). The PNM consisted of sodium polynucleotide (25 mg/mL), hyaluronic acid (10 mg/mL), lidocaine hydrochloride (3 mg/mL), glycerin (5 mg/mL), and GSH (1 mg/mL). The detailed compositions are listed in Table S1.

The PN used in the PN and PNM formulations was extracted from homogenized salmon testis tissue. The homogenate was adjusted to pH 5.5 to facilitate enzymatic processing for protein removal. After filtration-based protein separation, the extracted nucleic acids underwent precipitation and purification steps to isolate purified PN. The molecular size of the resulting PN was controlled to 400 ± 50 base pairs. The purity was confirmed by measuring the 260/280 absorbance ratio, which was 1.9, indicating high-purity nucleic acid.

### 4.2. Cell Culture

The keratinocyte cell line (HaCaT) used in this study was kindly provided by Professor Jeonghee Hong’s laboratory at Gachon University (Incheon, Republic of Korea). HaCaT cells were cultured in Dulbecco’s Modified Eagle Medium (DMEM; HyClone, Cytiva Logan, UT, USA) supplemented with 10% fetal bovine serum (FBS; HyClone) and 1% penicillin/streptomycin (P/S; Welgene, Gyeongsan, Republic of Korea) at 37 °C in a humidified atmosphere containing 5% CO_2_. Subculturing was performed using TrypLE (Gibco, Thermo Fisher Scientific, Waltham, MA, USA) when the cells reached approximately 70–80% confluence.

The melanoma cell line (B16F10; Korean Cell Line Bank, Seoul, Republic of Korea) was maintained in DMEM (HyClone) supplemented with 10% FBS and 1% P/S under the same culture conditions. B16F10 cells were passaged using TrypLE when they reached 80–90% confluence.

### 4.3. Experimental Design for AM, PN, or PNM Treatment In Vitro

To investigate the paracrine effects of AM, PN, and PNM on melanogenesis and keratinocyte response, a two-step co-culture model using HaCaT and B16F10 cells was established.

#### 4.3.1. Establishment of a UVB Exposure Model for Keratinocytes

For UVB irradiation, HaCaT cells were washed and covered with Dulbecco’s phosphate-buffered saline (DPBS; Gibco) to minimize light absorption by the culture medium, and then exposed to UVB light (peak wavelength: 306 nm; SANYO, Osaka, Japan). Following UVB exposure, DPBS was replaced with fresh growth medium.

To determine the treatment concentration, PNM was applied to HaCaT cells at concentrations ranging from 0 to 6.6 mg/mL. Based on the results of the GSH:GSSG ratio assay, a PNM concentration of 2.2 mg/mL was selected for subsequent experiments.

The PNM (2.2 mg/mL) is composed of PN (1.4 mg/mL) and AM (0.8 mg/mL). Each component was also tested individually at the same concentrations present in the PNM formulation for comparison. Non-irradiated and irradiated control groups were treated with DPBS only, without any active substances. After 48 h, culture supernatants (conditioned medium, CM) were collected and centrifuged at 300× *g* for 5 min to remove cell debris. Cell lysates were harvested for protein analysis.

#### 4.3.2. Establishment of CM-Treated Model for Melanoma

To assess the effects of keratinocyte-derived factors on melanogenesis, B16F10 mouse melanoma cells were cultured under standard conditions and then treated with CM collected from HaCaT cells pretreated with AM, PN, PNM, or the DPBS control for 48 h. After incubation, B16F120 cell lysates were harvested for analyses of tyrosinase activity, melanin content, and protein expression. This interspecies co-culture approach, although heterologous, enables modeling of keratinocyte-to-melanocyte paracrine interactions and is widely accepted in pigmentation research [50,51].

### 4.4. Animal Studies

All animal experiments were conducted in accordance with the guidelines of the Institutional Animal Care and Use Committee (IACUC) of Gachon University (approval number: LCDI-2023-0095) and complied with the ethical principles of the Association for Assessment and Accreditation of Laboratory Animal Care International (AAALAC International). No animals were excluded from the study. All experimental procedures and analyses included all assigned animals.

#### 4.4.1. Mouse Model and Maintenance

Six-week-old female HRM-2 mice were obtained from the Central Laboratory Animal Center (Incheon, Republic of Korea). Mice were housed under specific pathogen-free conditions in a temperature- and humidity-controlled environment (20–24 °C, 45–55% humidity) with a 12 h light/dark cycle and ad libitum access to standard chow and water. The mice were acclimatized for 2 w prior to the experiments. Age, sex, and strain of the animals were selected to ensure consistency in skin pigmentation responses. No additional inclusion or exclusion criteria were applied during group selection.

#### 4.4.2. Experimental Design for PNM or PN Injections in UVB-Induced Pigmentation Animal Model

Mice were randomly assigned to one of four groups (*n* = 5/group), three of which received UVB irradiation as described previously [52,53]. Briefly, UVB exposure was performed using a UVB lamp (peak wavelength: 306 nm; SANYO) at a dose of 200 mJ/cm^2^ every other day for 10 d, followed by daily exposure for 3 d. Saline, PNM, or PN (200 μL total volume) was administered via intradermal injection to the dorsal skin (2 × 2 cm regions) using a 27G screw needle. The treatments were administered once daily for 28 d. Skin samples were collected for subsequent analyses after the final treatment. All treatments and assessments were conducted by investigators who were blinded to the group allocations during outcome measurements.

#### 4.4.3. Skin Brightness Assessment

Skin brightness (L* value) was assessed using a CR-10 colorimeter (Konica Minolta Sensing, Inc., Osaka, Japan) calibrated in the CIELAB color space. Measurements were taken 42 d after initial UVB exposure (28 d after treatment initiation). Ten readings were collected for each sample and averaged to obtain the final L* value for statistical analysis.

### 4.5. Sample Preparation

#### 4.5.1. Protein Isolation and Concentration Quantification

Protein isolation was performed using the EzRIPA buffer kit (ATTO Corporation, Tokyo, Japan), following the manufacturer’s protocol. For cell samples, the cells were washed with cold PBS, and 1 mL of RIPA buffer was added. The cells were scraped using a cell scraper to collect the homogenates. For the skin samples, approximately 75 mg of tissue was homogenized in 0.8 mL RIPA buffer. The samples were then sonicated for 10 cycles of 40 s on/60 s off to break down the tissue, followed by incubation on ice for 10 min to enhance protein solubilization. The homogenates were then centrifuged at 14,000× *g* for 15 min at 4 °C to separate the proteins from the debris. Protein concentrations were quantified using a bicinchoninic acid (BCA) assay kit (Thermo Fisher Scientific) in accordance with the manufacturer’s instructions.

#### 4.5.2. Preparation of Paraffin-Embedded Skin Tissue Blocks

The skin tissues were fixed in 4% paraformaldehyde at 20–25 °C for 48 h and subsequently washed with distilled water. The fixed tissues were dehydrated using a graded ethanol series (Duksan, Ansan, Republic of Korea), cleared in xylene (Duksan), and embedded in paraffin (Leica). Paraffin blocks were sectioned into 7 µm thick slices using a microtome (Leica, Wetzlar, Germany), mounted on coated slides (Muto Pure Chemicals Co., Ltd., Tokyo, Japan), and incubated overnight at 60 °C for subsequent histological analyses.

### 4.6. Cell Viability Assessment

The cytotoxicity of PNM in keratinocytes was assessed using a Cell Counting Kit (CCK; TransGen Biotech, Beijing, China). Keratinocytes were seeded in 96-well plates at a density of 1 × 10^4^ cells per well and allowed to grow until 100% confluence. Once confluent, the cells were treated with various concentrations of PNM (0, 0.044, 0.44, 2.2, 4.4, 13.2, and 22 mg/mL) for 24 h. The medium was then removed, and the cells were washed with DPBS. A mixture of 10 µL of CCK reagent and 90 µL of basal medium was added to each well, and the plates were incubated at 37 °C for 2 h. Optical density was measured at 450 nm using a microplate reader (Thermo Fisher Scientific) to determine cell viability.

### 4.7. Measurement of GSH:GSSG Ratio and Tyrosinase Activity

The GSH:GSSG ratio in keratinocytes and mouse skin tissue was assessed using a GSH/GSSG-Glo assay kit (Promega, Madison, WI, USA). Tyrosinase activity in B16F10 cells and mouse skin tissue was measured using commercially available assay kits (Abcam, Cambridge, UK), according to the manufacturer’s instructions.

### 4.8. Western Blot Analysis

Protein samples (30 μg) from cell or skin samples were mixed with 4× LDS sample buffer and 10× reducing agent (Thermo Fisher Scientific). The protein mixtures were denatured by heating at 70 °C for 10 min and subjected to 10% sodium dodecyl sulfate-polyacrylamide gel electrophoresis using MOPS buffer (Invitrogen, Thermo Fisher Scientific) at 200 V for 25 min. A pre-stained protein molecular weight marker (EasySee Wesetern marker; TransGen Biotech) was loaded alongside the samples to estimate the molecular weights of the protein bands. Separated proteins were transferred onto polyvinylidene fluoride membranes (Millipore, Burlington, MA, USA) using a semi-dry transfer system (ATTO) at 1A for 10 min. To block nonspecific binding, the membranes were incubated in 5% skim milk (LPS solution, Daejeon, Republic of Korea) prepared in Tris-buffered saline with 0.1% Tween 20 (TTBS; SPL, Pocheon, Republic of Korea) on a shaker at room temperature for 1 h. After blocking, the membranes were washed three times with 0.1% TTBS and incubated overnight on a shaker at 4 °C with appropriately diluted primary antibodies (Table S3). After three washes with 0.1% TTBS, the membranes were incubated with horseradish peroxidase (HRP)-conjugated secondary antibodies (1:5000; Vector Laboratories, Burlingame, CA, USA) on a shaker at room temperature for 1 h. Protein bands were visualized using enhanced chemiluminescence substrates and imaged using a ChemiDoc imaging system (Bio-Rad, Hercules, CA, USA). Band intensities were quantified using ImageJ software (version 1.53s, NIH, Bethesda, MD, USA). β-actin bands were used as a loading control, and the results for each group were compared to the group corresponding to the first bar.

### 4.9. Enzyme-Linked Immunosorbent Assay (ELISA)

Microplates were coated with 100 nM carbonate–bicarbonate buffer (pH 9.6; Sigma-Aldrich, St. Louis, MO, USA) and incubated overnight at 4 °C. Unbound material was removed by washing the plates three times with phosphate-buffered saline (PBS) containing 0.1% Tween 20 (TPBS). To block nonspecific protein binding, the plates were incubated with 5% skim milk (LPS Solution) in 0.1% TPBS at 4 °C overnight. After blocking, the plates were washed three times with 0.1% TPBS, and 50 μg of protein samples was added to each well, followed by overnight incubation at 4 °C. The wells were then washed with 0.1% TPBS and incubated with primary antibodies diluted in PBS at 4°C overnight (details of the antibodies are provided in Table S3). After washing with TPBS, HRP-conjugated secondary antibodies (1:1000; Vector Laboratories) were added to the wells and incubated at room temperature for 4 h. To visualize the reaction, tetramethylbenzidine substrate solution (Sigma-Aldrich) was added to each well and incubated at room temperature for 15–20 min. The reaction was stopped by adding 2N sulfuric acid (Sigma-Aldrich). The absorbance was measured at 450 nm using a microplate reader (Thermo Fisher Scientific).

### 4.10. Staining

#### 4.10.1. Immunocytochemistry

HaCaT cells were seeded into eight-well plates at a density of 2.0 × 10^4^ cells per well. Once the cells were attached, all wells, except the non-UVB/PBS group, were exposed to UVB radiation (30 s, 306 nm peak wavelength, UV lamp; Sankyo) in the presence of DPBS, as described in Section 4.3.1. After UVB irradiation, DPBS, PNM, or PN was added to the wells, and the cells were incubated for 48 h. After incubation, the cells were washed with PBS and treated with 0.1% Triton X-100 (Sigma-Aldrich) for 5 min to permeabilize the cell membrane. The wells were washed three times with PBS for 10 min each. To block nonspecific binding, the cells were incubated with a serum blocking solution (Vector Laboratories) at room temperature for 1 h. The plates were then incubated overnight at 4 °C with primary antibodies targeting NF-κB (Table S3). After an overnight incubation, the plates were washed with PBS and incubated with secondary antibodies, Alexa Fluor 488 (Invitrogen, Thermo Fisher Scientific), at room temperature for 1 h. After another PBS wash, the nuclei were stained with DAPI (1 mg/mL; Sigma-Aldrich) for 30 s, washed with PBS, and mounted using Vectashield mounting medium (Vector Laboratories) to preserve fluorescence. Analysis was performed using confocal microscopy (LSM-710; Carl Zeiss, Oberkochen, Germany) at the core facility for cell to in vivo imaging.

#### 4.10.2. Immunohistochemistry

Paraffin-embedded skin tissue sections were deparaffinized and rehydrated through sequential immersion in xylene and a graded ethanol series (100–70%). Endogenous peroxidase activity was quenched by incubating the sections in 0.3% hydrogen peroxide (H_2_O_2_; Sigma-Aldrich) in methanol (Duksan) for 30 min. After three washes with PBS (5 min each), permeabilization was performed by incubating the sections in PBS containing 0.5% Triton X-100 for 5 min. Following another three washes with PBS (5 min each), nonspecific binding was blocked by incubating the sections with a serum blocking solution at room temperature for 1 h. The slides were then incubated overnight at 4 °C with primary antibodies (Table S3). After overnight incubation, slides were washed with PBS and incubated with biotin-conjugated secondary antibodies (Vector Laboratories) for 1 h at room temperature. The slides were subsequently washed with PBS, incubated with the ABC reagent (Vector Laboratories), and washed again. Color development was achieved by incubating the sections in 3,3′-diaminobenzidine (DAB) solution (Sigma-Aldrich) for 30 s to produce a brown color reaction. For counterstaining, slides were incubated in hematoxylin (KPNT, Cheongju, Republic of Korea) for 30 s, washed with distilled water, dehydrated using graded ethanol, and mounted with DPX mount solution (Sigma-Aldrich). The stained tissues were scanned using a slide scanner (Motic Scan Infinity 100; Motic, Beijing, China), and random images were captured for analysis. Protein expression was quantified using ImageJ software (version 1.53s, NIH). Positive staining was identified by the transition of color from yellow to brown, with the brown color extracted and converted to black for intensity quantification. The results for each group were compared with those of the non-UVB/saline samples.

#### 4.10.3. Fontana–Masson Staining

Fontana–Masson staining was performed according to the manufacturer’s protocol (Scytek, Logan, UT, USA). Briefly, paraffin-embedded skin tissue sections were deparaffinized and rehydrated through sequential immersion in xylene and a graded ethanol series (100–70%). The sections were then incubated in preheated Fontana ammonia silver solution at 60°C for 60 min. After incubation, the sections were washed with distilled water for 5 min. Non-melanin-stained areas were cleared using a 0.2% gold chloride solution and 5% sodium thiosulfate solution. Nuclei were counterstained with Nuclear Fast Red solution and the sections were dehydrated using graded ethanol. Finally, the sections were mounted using DPX mount solution (Sigma-Aldrich). The stained tissues were scanned using a slide scanner (Motic Scan Infinity 100), and random images were captured for analysis. Melanin content was quantified using ImageJ software (version 1.53s, NIH). Positive staining was identified as black regions extracted from the images, which were quantified based on their intensity. The results for each group were compared with those of the non-UVB/saline samples.

### 4.11. Melanin Amount

To evaluate the effects of PNM on melanogenesis, B16F10 cells were seeded into 24-well plates at a density of 1.0 × 10^5^ cells per well. After 24 h, the cells were treated for 48 h with the keratinocyte-CM prepared as described in Section 4.3. After the treatment period, the medium was removed, and the cells were washed with DPBS. The cells were detached via trypsinization, collected through centrifugation at 300× *g* 5 min, and washed again with DPBS. The cell pellets were dissolved directly in 500 μL of 1 N NaOH containing 10% DMSO (Sigma-Aldrich) and incubated at 90 °C for 90 min to solubilize melanin. The resulting solution was transferred to a 96-well plate, and the melanin content was measured at 405 nm using a microplate spectrophotometer (Thermo Fisher Scientific).

### 4.12. Synergy Analysis Using the Bliss Independence Model

To evaluate whether the combined treatment of PN and AM in PNM exhibits synergistic effects beyond simple additivity, the Bliss independence model was applied [49]. The expected combined effect (*E_Bliss_*) was calculated as

*E_Bliss_* = *E_PN_* + *E_AM_* − (*E_PN_* × *E_AM_*)

where *E_PN_* and *E_AM_* represent the fractional effects of PN and AM alone, respectively, calculated as percentage inhibition relative to the UVB-irradiated control. If the observed effect of PNM exceeds *E_Bliss_* (*δ*), synergy is indicated.

### 4.13. Statistical Analysis

All experiments were conducted in triplicate to ensure reproducibility and statistical significance. The Kruskal–Wallis test was used to compare groups, followed by the Mann–Whitney U test for post hoc comparisons. Results are expressed as mean ± standard deviation (SD). All statistical analyses were conducted using SPSS software version 26 (IBM, Armonk, NY, USA). Statistical significance is indicated in the figure legend for each result.

## 5. Conclusions

**Figure 6:**
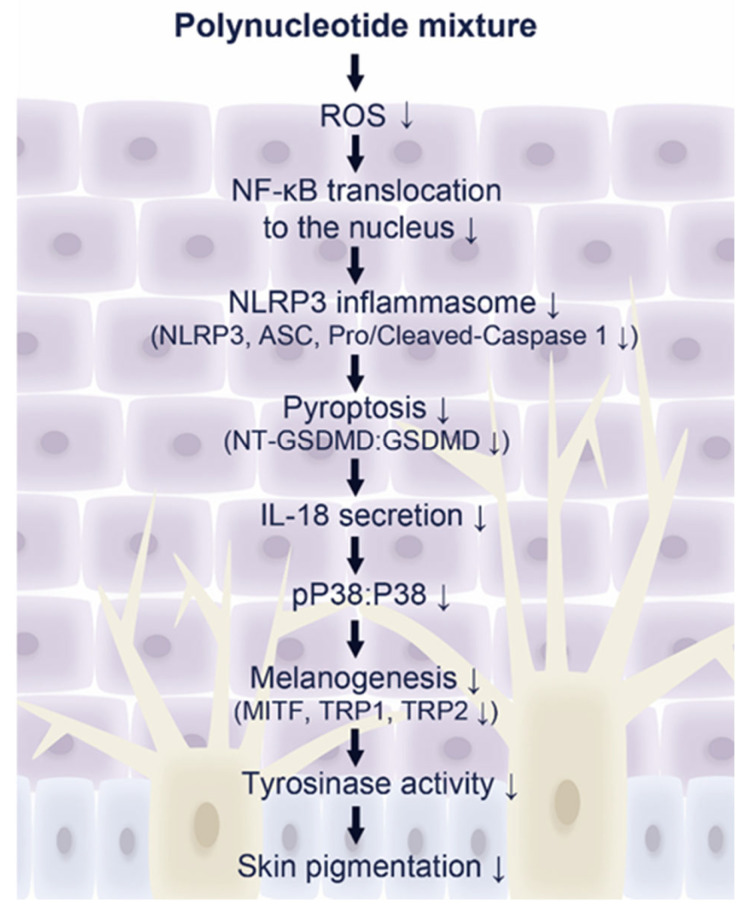
Proposed mechanism of PNM-mediated inhibition of UVB-induced hyperpigmentation. PNM treatment reduces ROS levels and suppresses NF-κB nuclear translocation in UVB-irradiated skin. This leads to an inhibition of NLRP3 inflammasome activation (decreased NLRP3, ASC, and cleaved/pro-caspase-1), thereby reducing pyroptosis (NT-GSDMD:GSDMD) and IL-18 secretion. Downstream, suppression of pP38:P38 signaling results in decreased melanogenesis-related pathways, including reduced expression of MITF, TRP1, TRP2, and tyrosinase activity, ultimately leading to an attenuation of skin pigmentation. ASC, apoptosis-associated speck-like protein containing a CARD; GSDMD, gasdermin D; IL-18, interleukin-18; MITF, microphthalmia-associated transcription factor; MW, molecular weight; NLRP3, NOD-, LRR- and pyrin domain-containing protein 3; NT-GSDMD, N-terminal GSDMD; pP38, phosphorylated P38; PN, polynucleotide; PNM, PN mixture; ROS, reactive oxygen species; SD, standard deviation; TRP, tyrosinase-related protein; UVB, ultraviolet B.

In conclusion, PN and PNM decreased oxidative stress and NF-κB activity, which was associated with decreased NLRP3 inflammasome formation and IL-18 in UVB- irradiated keratinocytes and animal skin. Moreover, these changes were associated with decreased melanogenesis-related signaling pathways of MITF, tyrosinase, TRP1, TRP2, and melanin content in UVB-irradiated skin (Figure 6). The anti-melanogenic effect of PNM was greater than that of PN. Furthermore, PN showed good biocompatibility and fewer complications than the other tyrosinase inhibitors. Thus, PN could be used to treat hyperpigmentation and increase collagen synthesis.

## Data Availability

All data are contained within the article.

## Supplementary Materials

The following supporting information can be downloaded at: https://www.mdpi.com/article/10.3390/ijms26136399/s1.

## References

[1] Wei M., He X., Liu N., Deng H. (2024). Role of reactive oxygen species in ultraviolet-induced photodamage of the skin. Cell Div..

[2] Lambeth J.D. (2004). NOX enzymes and the biology of reactive oxygen. Nat. Rev. Immunol..

[3] Moloney J.N., Cotter T.G. (2018). ROS signalling in the biology of cancer. Semin. Cell Dev. Biol..

[4] Zhai Y., Dang Y., Gao W., Zhang Y., Xu P., Gu J., Ye X. (2015). P38 and JNK signal pathways are involved in the regulation of phlorizin against UVB-induced skin damage. Exp. Dermatol..

[5] Hasegawa T., Nakashima M., Suzuki Y. (2016). Nuclear DNA damage-triggered NLRP3 inflammasome activation promotes UVB-induced inflammatory responses in human keratinocytes. Biochem. Biophys. Res. Commun..

[6] Agostini L., Martinon F., Burns K., McDermott M.F., Hawkins P.N., Tschopp J. (2004). NALP3 forms an IL-1beta-processing inflammasome with increased activity in Muckle-Wells autoinflammatory disorder. Immunity.

[7] Yang Y., Wang H., Kouadir M., Song H., Shi F. (2019). Recent advances in the mechanisms of NLRP3 inflammasome activation and its inhibitors. Cell Death Dis..

[8] Broz P., Dixit V.M. (2016). Inflammasomes: Mechanism of assembly, regulation and signalling. Nat. Rev. Immunol..

[9] Liu X., Zhang Z., Ruan J., Pan Y., Magupalli V.G., Wu H., Lieberman J. (2016). Inflammasome-activated gasdermin D causes pyroptosis by forming membrane pores. Nature.

[10] Ding J., Wang K., Liu W., She Y., Sun Q., Shi J., Sun H., Wang D.C., Shao F. (2016). Pore-forming activity and structural autoinhibition of the gasdermin family. Nature.

[11] Zhou J., Shang J., Song J., Ping F. (2013). Interleukin-18 augments growth ability of primary human melanocytes by PTEN inactivation through the AKT/NF-κB pathway. Int. J. Biochem. Cell Biol..

[12] Zhou J., Ling J., Wang Y., Shang J., Ping F. (2016). Cross-talk between interferon-gamma and interleukin-18 in melanogenesis. J. Photochem. Photobiol. B.

[13] Videira I.F., Moura D.F., Magina S. (2013). Mechanisms regulating melanogenesis. An. Bras. Dermatol..

[14] Brenner M., Hearing V.J. (2008). The protective role of melanin against UV damage in human skin. Photochem. Photobiol..

[15] Noh T.K., Chung B.Y., Kim S.Y., Lee M.H., Kim M.J., Youn C.S., Lee M.W., Chang S.E. (2016). Novel Anti-Melanogenesis Properties of Polydeoxyribonucleotide, a Popular Wound Healing Booster. Int. J. Mol. Sci..

[16] Squadrito F., Bitto A., Irrera N., Pizzino G., Pallio G., Minutoli L., Altavilla D. (2017). Pharmacological Activity and Clinical Use of PDRN. Front. Pharmacol..

[17] Khan A., Wang G., Zhou F., Gong L., Zhang J., Qi L., Cui H. (2022). Polydeoxyribonucleotide: A promising skin anti-aging agent. Chin. J. Plast. Reconstr. Surg..

[18] Irrera N., Bitto A., Vaccaro M., Mannino F., Squadrito V., Pallio G., Arcoraci V., Minutoli L., Ieni A., Lentini M. (2020). PDRN, a Bioactive Natural Compound, Ameliorates Imiquimod-Induced Psoriasis through NF-κB Pathway Inhibition and Wnt/β-Catenin Signaling Modulation. Int. J. Mol. Sci..

[19] Kim Y.J., Kim M.J., Kweon D.K., Lim S.T., Lee S.J. (2020). Polydeoxyribonucleotide Activates Mitochondrial Biogenesis but Reduces MMP-1 Activity and Melanin Biosynthesis in Cultured Skin Cells. Appl. Biochem. Biotechnol..

[20] Kim T.H., Heo S.Y., Oh G., Heo S.J., Jung W.K. (2021). Applications of Marine Organism-Derived Polydeoxyribonucleotide: Its Potential in Biomedical Engineering. Mar. Drugs.

[21] Lee Y.J., Kim H.T., Lee Y.J., Paik S.H., Moon Y.S., Lee W.J., Chang S.E., Lee M.W., Choi J.H., Jung J.M. (2022). Comparison of the effects of polynucleotide and hyaluronic acid fillers on periocular rejuvenation: A randomized, double-blind, split-face trial. J. Dermatol. Treat..

[22] Kim J.H., Jeong J.J., Lee Y.I., Lee W.J., Lee C., Chung W.Y., Nam K.H., Lee J.H. (2018). Preventive effect of polynucleotide on post-thyroidectomy scars: A randomized, double-blinded, controlled trial. Lasers Surg. Med..

[23] Bitto A., Oteri G., Pisano M., Polito F., Irrera N., Minutoli L., Squadrito F., Altavilla D. (2013). Adenosine receptor stimulation by polynucleotides (PDRN) reduces inflammation in experimental periodontitis. J. Clin. Periodontol..

[24] Altavilla D., Bitto A., Polito F., Marini H., Minutoli L., Di Stefano V., Irrera N., Cattarini G., Squadrito F. (2009). Polydeoxyribonucleotide (PDRN): A safe approach to induce therapeutic angiogenesis in peripheral artery occlusive disease and in diabetic foot ulcers. Cardiovasc. Hematol. Agents Med. Chem..

[25] Yi K.H., Winayanuwattikun W., Kim S.Y., Wan J., Vachatimanont V., Putri A.I., Hidajat I.J., Yogya Y., Pamela R. (2024). Skin boosters: Definitions and varied classifications. Ski. Res. Technol..

[26] Zitka O., Skalickova S., Gumulec J., Masarik M., Adam V., Hubalek J., Trnkova L., Kruseova J., Eckschlager T., Kizek R. (2012). Redox status expressed as GSH:GSSG ratio as a marker for oxidative stress in paediatric tumour patients. Oncol. Lett..

[27] Valavanidis A., Vlachogianni T., Fiotakis C. (2009). 8-hydroxy-2′-deoxyguanosine (8-OHdG): A critical biomarker of oxidative stress and carcinogenesis. J. Environ. Sci. Health C Environ. Carcinog. Ecotoxicol. Rev..

[28] Hossain M.R., Ansary T.M., Komine M., Ohtsuki M. (2021). Diversified Stimuli-Induced Inflammatory Pathways Cause Skin Pigmentation. Int. J. Mol. Sci..

[29] Park H.Y., Kosmadaki M., Yaar M., Gilchrest B.A. (2009). Cellular mechanisms regulating human melanogenesis. Cell. Mol. Life Sci..

[30] Hida T., Kamiya T., Kawakami A., Ogino J., Sohma H., Uhara H., Jimbow K. (2020). Elucidation of Melanogenesis Cascade for Identifying Pathophysiology and Therapeutic Approach of Pigmentary Disorders and Melanoma. Int. J. Mol. Sci..

[31] Fang J., Ouyang M., Qu Y., Wang M., Huang X., Lan J., Lai W., Xu Q. (2022). Advanced Glycation End Products Promote Melanogenesis by Activating NLRP3 Inflammasome in Human Dermal Fibroblasts. J. Investig. Dermatol..

[32] Antonuccio P., Micali A.G., Romeo C., Freni J., Vermiglio G., Puzzolo D., Squadrito F., Irrera N., Marini H.R., Rana R.A. (2021). NLRP3 Inflammasome: A New Pharmacological Target for Reducing Testicular Damage Associated with Varicocele. Int. J. Mol. Sci..

[33] Bukhari S.N.A., Roswandi N.L., Waqas M., Habib H., Hussain F., Khan S., Sohail M., Ramli N.A., Thu H.E., Hussain Z. (2018). Hyaluronic acid, a promising skin rejuvenating biomedicine: A review of recent updates and pre-clinical and clinical investigations on cosmetic and nutricosmetic effects. Int. J. Biol. Macromol..

[34] Siquier-Dameto G., Boisnic S., Boadas-Vaello P., Verdú E. (2023). Anti-Aging and Depigmentation Effect of a Hyaluronic Acid Mechanically Stabilized Complex on Human Skin Explants. Polymers.

[35] Onodera Y., Teramura T., Takehara T., Fukuda K. (2015). Hyaluronic acid regulates a key redox control factor Nrf2 via phosphorylation of Akt in bovine articular chondrocytes. FEBS Open Bio.

[36] Sonthalia S., Daulatabad D., Sarkar R. (2016). Glutathione as a skin whitening agent: Facts, myths, evidence and controversies. Indian. J. Dermatol. Venereol. Leprol..

[37] Villarama C.D., Maibach H.I. (2005). Glutathione as a depigmenting agent: An overview. Int. J. Cosmet. Sci..

[38] Pillaiyar T., Manickam M., Namasivayam V. (2017). Skin whitening agents: Medicinal chemistry perspective of tyrosinase inhibitors. J. Enzyme Inhib. Med. Chem..

[39] Shivhare S.C., Malviya K.G., Shivhare Malviya K.K., Jain V. (2013). A review: Natural skin lighting and nourishing agents. Res. J. Top. Cosmet. Sci..

[40] Fisher A.A. (1983). Current contact news. Hydroquinone uses and abnormal reactions. Cutis.

[41] Zhou H., Kepa J.K., Siegel D., Miura S., Hiraki Y., Ross D. (2009). Benzene metabolite hydroquinone up-regulates chondromodulin-I and inhibits tube formation in human bone marrow endothelial cells. Mol. Pharmacol..

[42] Fujimoto N., Onodera H., Mitsumori K., Tamura T., Maruyama S., Ito A. (1999). Changes in thyroid function during development of thyroid hyperplasia induced by kojic acid in F344 rats. Carcinogenesis.

[43] Spínola V., Mendes B., Câmara J.S., Castilho P.C. (2013). Effect of time and temperature on vitamin C stability in horticultural extracts. UHPLC-PDA vs iodometric titration as analytical methods. LWT-Food Sci. Technol..

[44] Galeano M., Bitto A., Altavilla D., Minutoli L., Polito F., Calò M., Lo Cascio P., Stagno d’Alcontres F., Squadrito F. (2008). Polydeoxyribonucleotide stimulates angiogenesis and wound healing in the genetically diabetic mouse. Wound Repair. Regen..

[45] Squadrito F., Bitto A., Altavilla D., Arcoraci V., De Caridi G., De Feo M.E., Corrao S., Pallio G., Sterrantino C., Minutoli L. (2014). The effect of PDRN, an adenosine receptor A2A agonist, on the healing of chronic diabetic foot ulcers: Results of a clinical trial. J. Clin. Endocrinol. Metab..

[46] Cavallini M., Bartoletti E., Maioli L., Massirone A., Pia Palmieri I., Papagni M., Priori M., Trocchi G. (2021). Consensus report on the use of PN-HPT™ (polynucleotides highly purified technology) in aesthetic medicine. J. Cosmet. Dermatol..

[47] Kim J.H., Kwon T.R., Lee S.E., Jang Y.N., Han H.S., Mun S.K., Kim B.J. (2020). Comparative Evaluation of the Effectiveness of Novel Hyaluronic Acid-Polynucleotide Complex Dermal Filler. Sci. Rep..

[48] Krutmann J., Gilchrest B.A., Gilchrest B.A., Krutmann J. (2006). Photoaging of skin. Skin Aging.

[49] Zhao W., Sachsenmeier K., Zhang L., Sult E., Hollingsworth R.E., Yang H. (2014). A New Bliss Independence Model to Analyze Drug Combination Data. J. Biomol. Screen..

[50] Chen H.W., Chou Y.S., Young T.H., Cheng N.C. (2020). Inhibition of melanin synthesis and melanosome transfer by chitosan biomaterials. J. Biomed. Mater. Res. B Appl. Biomater..

[51] Yoon Y., Bae S., Kim T.J., An S., Lee J.H. (2023). Nodakenin Inhibits Melanogenesis via the ERK/MSK1 Signaling Pathway. Pharmazie.

[52] Byun K.A., Lee S.Y., Oh S., Batsukh S., Jang J.W., Lee B.J., Rheu K.M., Li S., Jeong M.S., Son K.H. (2024). Fermented Fish Collagen Attenuates Melanogenesis via Decreasing UV-Induced Oxidative Stress. Mar. Drugs.

[53] Byun K.A., Park Y., Oh S., Batsukh S., Son K.H., Byun K. (2024). Co-Treatment with Phlorotannin and Extracellular Vesicles from *Ecklonia cava* Inhibits UV-Induced Melanogenesis. Antioxidants.

