# Supplementary material for: Polynucleotide Mixture Attenuates Ultraviolet B-Induced Skin Pigmentation
Source: Int J Mol Sci. 2025 Jul 3;26(13):6399. doi: 10.3390/ijms26136399 (PMC12250556; doi:10.3390/ijms26136399)
Supplement: Supplementary file 1 [file ijms-26-06399-s001.zip › ijms-3692749-supplementary.pdf]

International Journal of *Molecular Sciences*

Article

Polynucleotide Mixture Attenuates Ultraviolet B-Induced Skin Pigmentation

Seyeon Oh [1], Hee-Dae Jeon [2], Nark-Kyoung Rho [3], Kuk Hui Son [4,*] and Kyunghee Byun [1,5,6,*]

1 Functional Cellular Networks Laboratory, Lee Gil Ya Cancer and Diabetes Institute, Gachon University, Incheon 21999, Republic of Korea
2 Banobagi Dermatology Clinic, Seoul 06109, Republic of Korea
3 Leaders Aesthetic Laser & Cosmetic Surgery Center, Seoul 06014, Republic of Korea
4 Department of Thoracic and Cardiovascular Surgery, Gachon University Gil Medical Center, Gachon University, Incheon 21565, Republic of Korea
5 Department of Anatomy & Cell Biology, College of Medicine, Gachon University, Incheon 21936, Republic of Korea
6 Department of Health Sciences and Technology, Gachon Advanced Institute for Health & Sciences and Technology (GAIHST), Gachon University, Incheon 21999, Republic of Korea
* Correspondence: dr632@gachon.ac.kr (K.H.S.); khbyun1@gachon.ac.kr (K.B.); Tel.: +82-32-460-3666 (K.H.S.); +82-32-899-6511 (K.B.)

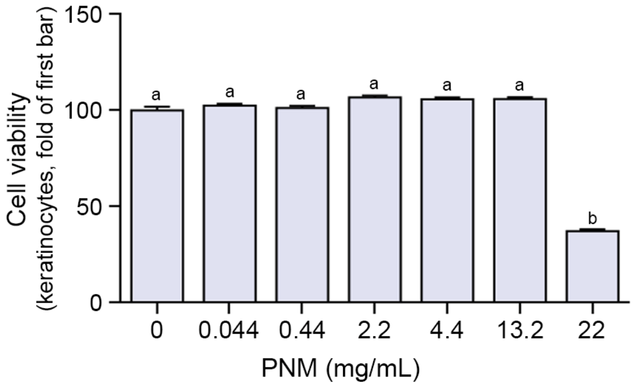

Figure S1. Effect of PNM on cell viability of keratinocyte in dose-dependent manner. Cell viability was in keratinocytes following treatment with various concentrations of PNM (0, 0.044, 0.44, 2.2, 4.4, 13.2, and 22 mg/mL). Data are expressed as a percentage of the untreated control (0 mg/mL). Results indicate that PNM did not significantly affect keratinocyte viability at lower concentrations, whereas a significant reduction was observed at the highest concentration (22 mg/mL). Values represent the mean ± SD of three independent experiments. Statistical comparisons were performed using the Mann–Whitney U test, with $p < 0.05$ considered significant. Identical letters (a,b) denote no statistically significant difference between the respective groups. PNM, polynucleotides mixture; SD, standard deviation.

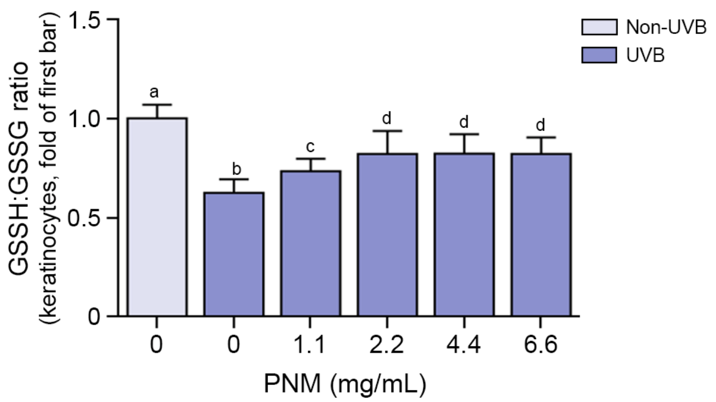

Figure S2. Effect on PNM on GSH:GSSG ratio in UVB-irradiated keratinocytes. The GSH:GSSG ratio was measured in keratinocytes treated with different concentrations of PNM (0,1.1, 2.2, 4.4, and 6.6 mg/mL) in the presence or absence of UVB irradiation. The non-UVB control group (first bar) was set as 1-fold. UVB irradiation significantly reduced the GSH:GSSG ratio. Treatment with PNM increased the GSH:GSSG ratio in UVB-irradiated keratinocytes in a dose-dependent manner. Data are presented as the mean ± SD from three independent experiments. Statistical analysis was conducted using the Mann–Whitney U test, and differences were considered significant at $p < 0.05$. Groups sharing the same letter (a–d) are not significantly different. GSH, glutathione; GSSG, glutathione disulfide; PNM, polynucleotides mixture; SD, standard deviation; UVB, ultraviolet B.

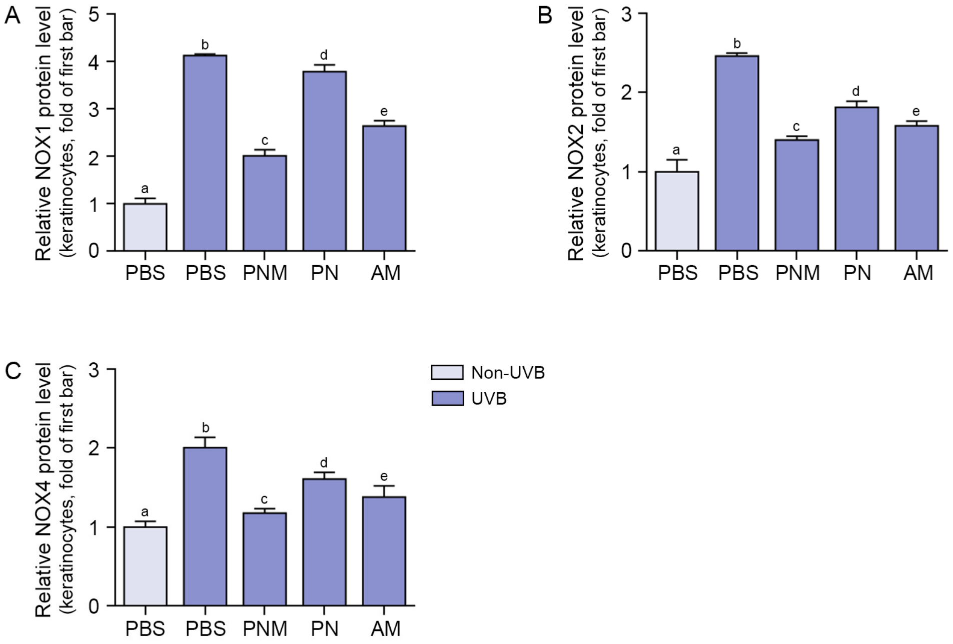

Figure S3. Reduction of NOX protein expression by PNM and PN in UVB-irradiated keratinocytes. Relative protein expression levels of (**A**) NOX1, (**B**) NOX2, and (**C**) NOX4 in keratinocytes exposed to UVB and treated with PNM or PN. These graphs quantify the Western blot results of Figure 1A. UVB irradiation significantly increased NOX1/2/4 expression levels compared to the NON-UVB/PBS (first bar). Treatment with PNM or PN significantly reduced the UVB-induced increase in NOX protein levels. Protein expression levels were normalized to β-actin and are expressed as fold changes relative to the NON-UVB/PBS (first bar). Results are shown as mean ± SD from three separate experiments. Group comparisons were analyzed using the Mann–Whitney U test, with statistical significance defined as $p < 0.05$. The same letters (a–e) indicate groups with no significant differences. AM, antioxidant mixture; NOX, NADPH oxidase; PBS, phosphate-buffered saline; PN, polynucleotides; PNM, PN mixture; SD, standard deviation; UVB, ultraviolet B.

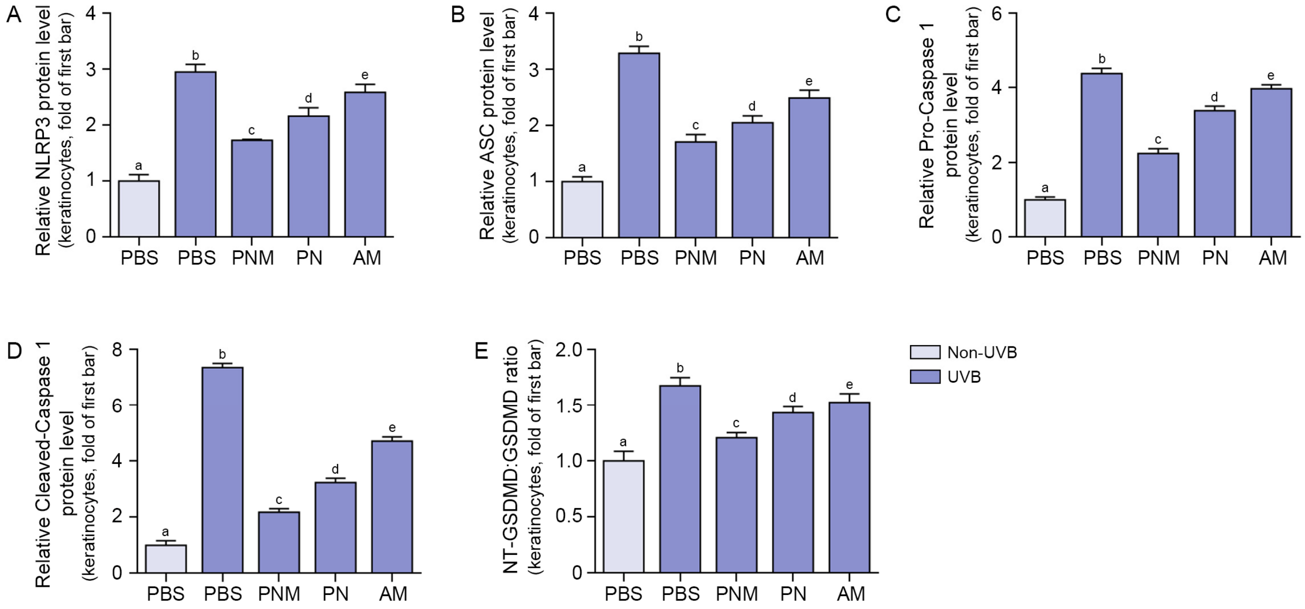

Figure S4. Suppression of NLRP3 inflammasome activation and pyroptosis by PNM or PN in UVB-irradiated keratinocytes. Quantification of NLRP3 inflammasome-related protein levels in keratinocytes exposed to UVB and treated with PNM or PN. These graphs represent the analysis results of the Western blot images shown in Figure 2A and B. (**A**) Relative NLRP3 protein levels, showing a significant increase after UVB exposure, which was reduced by PNM or PN treatment. (**B**) Relative ASC protein levels, demonstrating that PNM or PN treatment mitigated the UVB-induced increase. (**C**) Relative pro-caspase-1 protein levels, indicating that UVB-induced upregulation was reduced by PNM or PN treatment. (**D**) Relative cleaved-caspase-1 protein levels, showing that PNM or PN suppressed UVB-induced inflammasome activation. (**E**) NT-GSDMD:GSDMD ratio, representing pyroptosis activation, which was reduced by PNM or PN treatment. Protein expression levels were normalized to β-actin and are expressed as fold changes relative to the NON-UVB/PBS (first bar). Each value represents the mean ± SD of three biologically independent replicates. Statistical significance was evaluated by the Mann–Whitney U test, with $p < 0.05$ regarded as significant. Letters (a–e) indicate statistically indistinguishable groups. AM, antioxidant mixture; ASC, apoptosis-associated speck-like protein containing a CARD; GSDMD, gasdermin D; NLRP3, NOD-, LRR- and pyrin domain-containing protein 3; NT-GSDMD, N-terminal GSDMD; PBS, phosphate-buffered saline; PN, polynucleotides; PNM, PN mixture; SD, standard deviation; UVB, ultraviolet B.

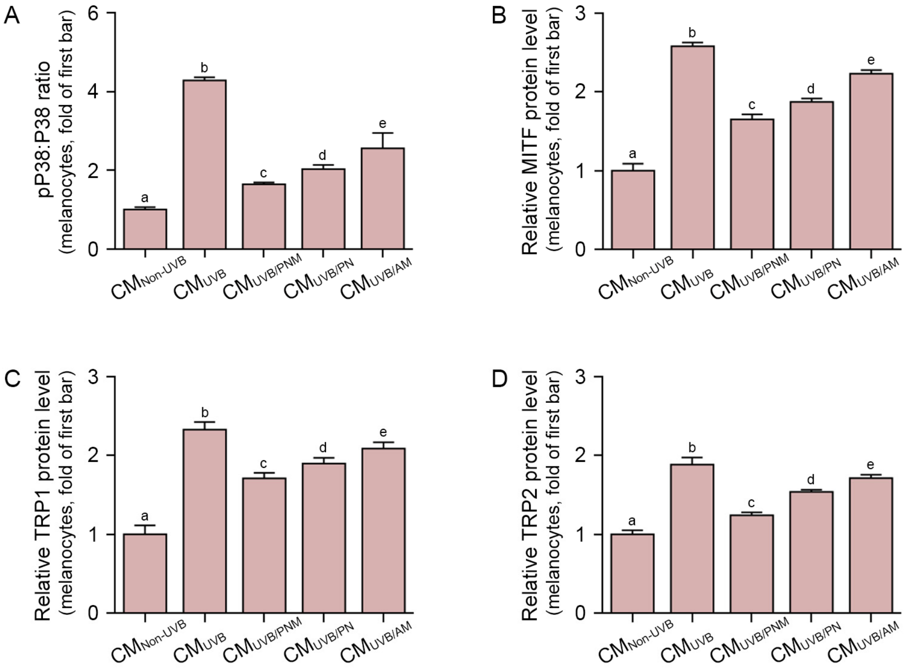

Figure S5. Reduction of melanogenesis-related protein expression by PNM or PN in melanocyte treated CM from UVB-irradiated keratinocyte. Quantification of Western blot results from Figure 2D, showing the effects of PNM and PN on melanogenesis-related protein expression in melanocytes treated with CM from Non-UVB- or UVB-irradiated keratinocytes. (**A**) The pP38:P38 ratio was significantly increased in the CM$_{UVB}$ group, while CM$_{UVB/PNM}$ or CM$_{UVB/PN}$ treatment reduced this elevation. (**B**) MITF protein levels were upregulated by CM$_{UVB}$ treatment, and CM$_{UVB/PNM}$ or CM$_{UVB/PN}$ treatment attenuated this effect. (**C,D**) TRP1 (C) and TRP2 (D) protein expression levels were significantly increased in CM$_{UVB}$-treated melanocytes, and CM$_{UVB/PNM}$ or CM$_{UVB/PN}$ treatment led to a reduction in their expression. Protein expression levels were normalized to β-actin and expressed as fold changes relative to the CM$_{Non\text{-}UVB}$ group (first bar). Each value represents the mean ± SD of three biologically independent replicates. Group comparisons were analyzed using the Mann–Whitney U test, with statistical significance defined as $p < 0.05$. The same letters (a–e) indicate groups with no significant differences. AM, antioxidant mixture; CM, conditioned media from keratinocyte; CM$_{Non\text{-}UVB}$, conditioned media from the Non-UVB/PBS group of keratinocytes; CM$_{UVB}$, conditioned media from the UVB/PBS group of keratinocytes; CM$_{UVB/PN}$, conditioned media from the UVB/PN group of keratinocytes; CM$_{UVB/PNM}$, conditioned media from the UVB/PNM group of keratinocytes; MITF, microphthalmia-associated transcription factor; PBS, phosphate-buffered saline; pP38, phosphorylated P38; PN, polynucleotides; PNM, PN mixture; SD, standard deviation; TRP, tyrosinase-related protein; UVB, ultraviolet B.

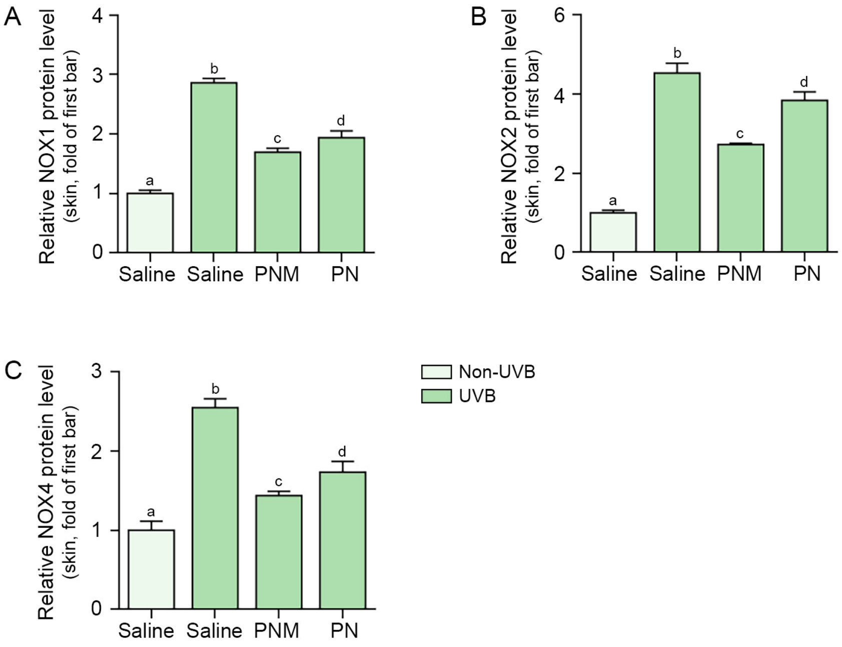

Figure S6. Attenuation of UVB-induced NOX protein expression by PNM and PN in mouse skin. (**A–C**) Relative protein expression levels of NOX1 (A), NOX2 (B), an NOX4 (C) were analyzed in mouse skin following UVB irradiation and treatment with PNM or PN. UVB exposure significantly upregulated NOX protein expression compared to the NON-UVB/Saline, while PNM or PN treatment effectively mitigated this increase. These results correspond to the Western blot data shown in Figure 3A. Protein expression levels were normalized to β-actin and are presented as fold changes relative to the Non-UVB/Saline group (first bar). Data are expressed as mean ± SD from three independent biological replicates. Statistical significance was assessed using the Mann–Whitney U test ($p < 0.05$). Groups sharing the same letter (a–d) are not significantly different. NOX, NADPH oxidase; PN, polynucleotides; PNM, PN mixture; SD, standard deviation; UVB, ultraviolet B.

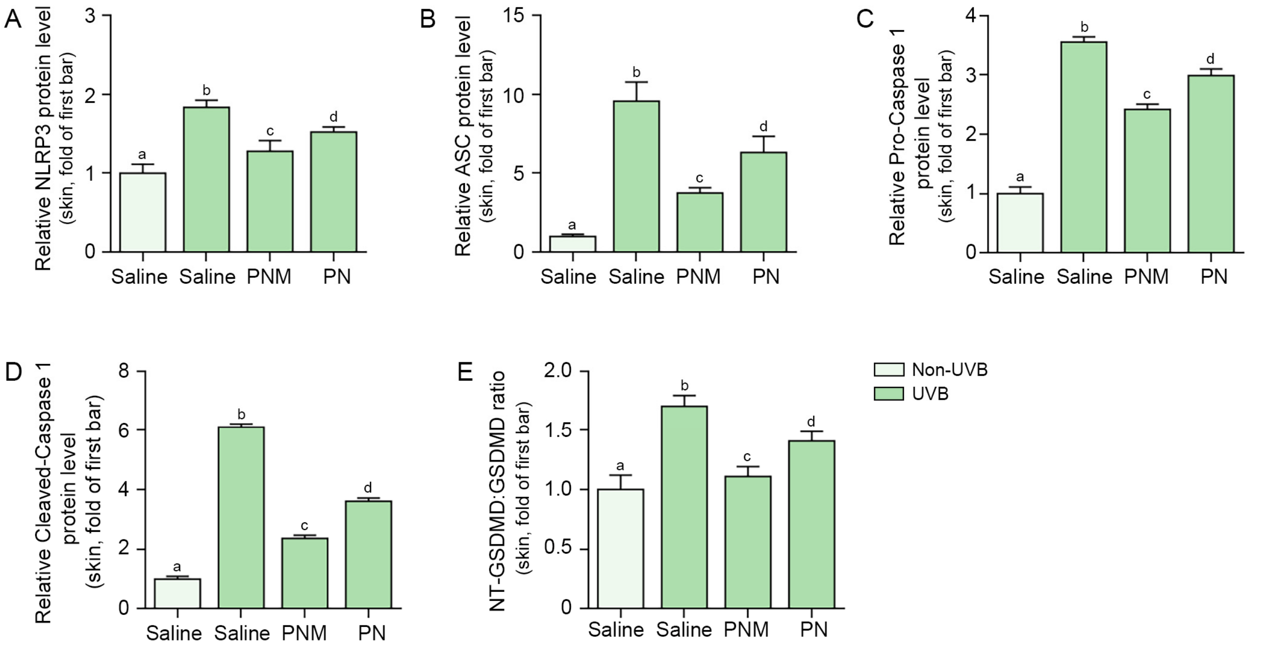

Figure S7. Inhibition of UVB-induced NLRP3 inflammasome activation and pyroptosis by PNM or PN in mouse skin. Analysis of NLRP3 inflammasome-related protein expression in UVB-irradiated skin treated with PNM or PN. These graphs represent the quantification of Western blot images shown in Figure 4A and B. (**A**) NLRP3 protein expression was significantly increased after UVB exposure and was attenuated by PNM or PN treatment. (**B**) ASC protein levels were elevated in the UVB-irradiated group, and treatment with PNM or PN reduced this increase. (**C**) Pro-caspase-1 expression was upregulated following UVB exposure, but PNM or PN treatment effectively suppressed this induction. (**D**) Cleaved-caspase-1 levels, a marker of inflammasome activation, were significantly increased in UVB-exposed skin, while PNM or PN treatment alleviated this effect. (**E**) NT-GSDMD:GSDMD ratio, an indicator of pyroptosis activation, was significantly higher in the UVB-exposed group but was reduced by PNM or PN treatment. Protein expression levels were normalized to β-actin and are presented as fold changes relative to the Non-UVB/Saline group (first bar). Values are presented as the mean ± SD from three biologically independent experiments. Group differences were evaluated using the Mann–Whitney U test, with $p < 0.05$ considered statistically significant. Identical letters (a–d) indicate no significant differences between groups. ASC, apoptosis-associated speck-like protein containing a CARD; GSDMD, gasdermin D; NLRP3, NOD-, LRR- and pyrin domain-containing protein 3; NT-GSDMD, N-terminal GSDMD; PBS, phosphate-buffered saline; PN, polynucleotides; PNM, PN mixture; SD, standard deviation; UVB, ultraviolet B.

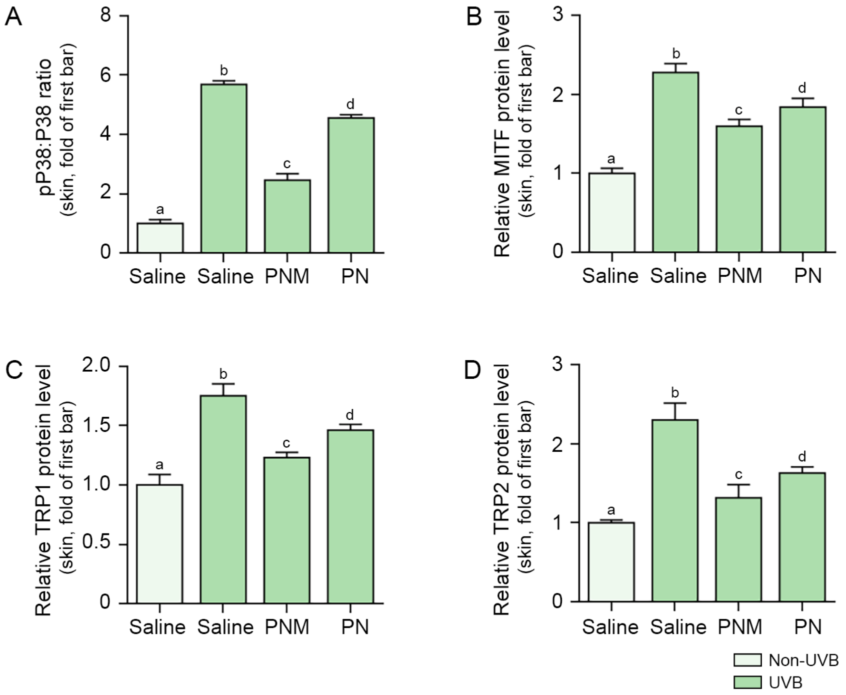

Figure S8. Attenuation of UVB-induced melanogenesis-related protein expression by PNM or PN in mouse skin. Analysis of melanogenesis-related protein expression in UVB-exposed skin treated with PNM or PN. These graphs represent the quantification of Western blot results shown in Figure 5A. (**A**) The pP38:P38 ratio was significantly increased following UVB exposure, while PNM or PN treatment reduced this elevation. (**B**) MITF protein levels were upregulated by UVB exposure, and treatment with PNM or PN effectively mitigated this increase. (**C**,**D**) TRP1 (C) and TRP2 (D) protein expression levels were significantly increased in UVB-exposed skin, but PNM or PN treatment suppressed their expression. Protein expression levels were normalized to β-actin and expressed as fold changes relative to the Non-UVB/Saline group (first bar). Each result represents the mean ± SD of three independent experiments. The Mann–Whitney U test was used for group comparisons, and $p < 0.05$ was considered statistically significant. Groups sharing the same letter (a–d) were not significantly different. MITF, microphthalmia-associated transcription factor; pP38, phosphorylated P38; PN, polynucleotides; PNM, PN mixture; SD, standard deviation; TRP, tyrosinase-related protein; UVB, ultraviolet B.

Table S1. Compositions of PNM, PN and AM.

Components	Contents (mg/mL)		
	PNM	PN	AM
**Sodium polynucleotide**	25	25	
**Hyaluronic acid**	10		10
**Lidocaine hydrochloride**	3	3	
**Glycerin**	5		5
**GSH**	1		1

The table presents the components of PNM, PN and AM. The detailed component lists and their respective concentrations are listed in the table. AM, antioxidant mixture; GSH, Glutathione; PN, polynucleotides; PNM, PN mixture.

Table S2. Synergistic effect (δ) of combined PN and Am treatment evaluated by Bliss independence model.

	**Inhibition (%)**		**Expected (%)**	**Observed (%)**	**δ (Synergy)**
	**PN**	**PNM**	**PNM**	**PNM**	
**NOX1**	8.26	35.96	41.25	51.24	9.99
**NOX2**	26.28	35.78	52.65	53.19	0.54
**NOX4**	17.39	30.19	42.33	44.25	1.92
**GSH:GSSG**	43.06	61.59	78.13	91.54	13.41
**8-OHdG**	15.22	19.70	31.92	34.19	2.27
**NF-κB**	25.31	34.78	51.30	56.45	5.15
**NLRP3**	26.75	12.39	35.83	41.40	5.57
**ASC**	37.56	21.25	50.83	51.05	0.22
**Pro-Caspase 1**	22.76	9.30	29.95	48.78	18.83
**Cleave-Caspase 1**	53.26	33.20	68.78	73.14	4.36
**NT-GSDMD:GSDMD ratio**	14.50	9.04	22.23	27.90	5.67
**IL-18**	28.24	17.57	40.84	46.31	5.47
**pP38:P38 ratio**	33.85	21.80	48.27	51.93	3.66
**MITF**	27.43	5.78	31.62	37.96	6.34
**TRP1**	18.42	10.23	26.76	28.65	1.89
**TRP2**	18.29	9.03	25.67	34.07	8.40
**Tyrosinase activity**	16.73	5.98	21.71	34.46	12.75
**Melanin amount**	22.22	10.18	30.13	36.64	6.51

Table S3. List of antibodies used for western blot, ELISA, ICC and IHC.

Antibody	Dilution rate			
	Western blot	ELISA	ICC	IHC
**ASC**	1:500			
**β-actin**	1:1,000			
**Cleaved-Caspas-1**	1:1,000			
**GSDMD**	1:1,000			
**IL-18**		1:200		
**MITF**	1:500			
**NF-κB**			1:200	1:200
**NLRP3**	1:500			
**Nox1**	1:1,000			
**Nox2**	1:1,000			
**Nox4**	1:1,000			
**NT-GSDMD**	1:1,000			
**p38**	1:1,000			
**pp38**	1:1,000			
**Pro-Caspas-1**	1:1,000			
**TRP1**	1:500			
**TRP2**	1:500			
**8-OHdG**		1:500		

The table presents the list of antibodies used in this study for Western blot, ELISA, ICC and IHC. ASC, apoptosis-associated speck-like protein containing a CARD; ELISA, Enzyme-linked immunosorbent assay; GSDMD, gasdermin D; ICC, Immunocytochemistry; IHC, Immunohistochemistry; IL-18, interleukin-18; NF-κB, nuclear factor-kappa B; NLRP3, NOD-, LRR- and pyrin domain-containing protein 3; NOX, NADPH oxidases; NT-GSDMD, N-terminal GSDMD; pp38, phosphorylated p38; 8-OHdG, TRP, tyrosinase-related protein; 8-Hydroxy-2′-deoxyguanosine.
